# Altered Skeletal Muscle Fatty Acid Handling in Subjects with Impaired Glucose Tolerance as Compared to Impaired Fasting Glucose

**DOI:** 10.3390/nu8030164

**Published:** 2016-03-14

**Authors:** Gijs H. Goossens, Chantalle C. M. Moors, Johan W. E. Jocken, Nynke J. van der Zijl, Anneke Jans, Ellen Konings, Michaela Diamant, Ellen E. Blaak

**Affiliations:** 1 Department of Human Biology, NUTRIM School of Nutrition and Translational Research in Metabolism, Maastricht University Medical Centre^+^, Maastricht 6200 MD, The Netherlands; c.moors@maastrichtuniversity.nl (C.C.M.M.); j.jocken@maastrichtuniversity.nl (J.W.E.J.); A.jans@maastrichtuniversity.nl (A.J.); ellen.konings@maastrichtuniversity.nl (E.K.); e.blaak@maastrichtuniversity.nl (E.E.B.); 2 Diabetes Center, Department of Internal Medicine, VU University Medical Center, Amsterdam 1007 MB, The Netherlands; nj.vanderzijl@vumcl.nl (N.J.Z.); m.diamant@vumc.nl (M.D.)

**Keywords:** skeletal muscle, lipid metabolism, insulin resistance, impaired glucose tolerance, impaired fasting glucose

## Abstract

Altered skeletal muscle fatty acid (FA) metabolism contributes to insulin resistance. Here, we compared skeletal muscle FA handling between subjects with impaired fasting glucose (IFG; *n* = 12 (7 males)) and impaired glucose tolerance (IGT; *n* = 14 (7 males)) by measuring arterio-venous concentration differences across forearm muscle. [^2^H_2_]-palmitate was infused intravenously, labeling circulating endogenous triacylglycerol (TAG) and free fatty acids (FFA), whereas [U-^13^C]-palmitate was incorporated in a high-fat mixed-meal, labeling chylomicron-TAG. Skeletal muscle biopsies were taken to determine muscle TAG, diacylglycerol (DAG), FFA, and phospholipid content, their fractional synthetic rate (FSR) and degree of saturation, and gene expression. Insulin sensitivity was assessed using a hyperinsulinemic-euglycemic clamp. Net skeletal muscle glucose uptake was lower (*p* = 0.018) and peripheral insulin sensitivity tended to be reduced (*p* = 0.064) in IGT as compared to IFG subjects. Furthermore, IGT showed higher skeletal muscle extraction of VLDL-TAG (*p* = 0.043), higher muscle TAG content (*p* = 0.025), higher saturation of FFA (*p* = 0.004), lower saturation of TAG (*p* = 0.017) and a tendency towards a lower TAG FSR (*p* = 0.073) and a lower saturation of DAG (*p* = 0.059) *versus* IFG individuals. Muscle oxidative gene expression was lower in IGT subjects. In conclusion, increased liver-derived TAG extraction and reduced lipid turnover of saturated FA, rather than DAG content, in skeletal muscle accompany the more pronounced insulin resistance in IGT *versus* IFG subjects.

## 1. Introduction

Impairments in skeletal muscle fatty acid (FA) handling may contribute to the development of insulin resistance and type 2 diabetes. Insulin resistant conditions, as present in subjects with impaired fasting glucose (IFG) and impaired glucose tolerance (IGT) [1,2], are often characterized by increased circulating concentrations of triacylglycerol (TAG) and free fatty acids (FFA), which is partly due to impairments in adipose tissue lipid handling [3,4]. This results in an increased supply of FAs to non-adipose tissues, including the liver and skeletal muscle, which may lead to ectopic fat storage [4,5,6,7]. In addition, intrinsic disturbances in skeletal muscle FA handling, including an impaired mitochondrial lipid oxidation, may contribute to lipid accumulation [8,9,10] and subsequent insulin resistance [5,7,11]. Within the last decade, however, it has become clear that the amount of lipids *per se* do not determine insulin resistance. Rather, a complex interplay between FA supply, type of FA, muscle lipid turnover and the subcellular localization and composition of specific bioactive lipid metabolites is involved in muscle insulin resistance [5,7,11]. More specific, others and we have recently demonstrated that an increased saturation of FAs in skeletal muscle (membrane) DAG rather than an increased total DAG content was related to insulin resistance [12,13,14]. These findings are in line with studies in human muscle cell lines and rodents, showing that long-chain saturated FAs accumulate preferentially as DAG and relate to insulin resistance, whereas unsaturated FAs are more readily converted into TAG [15,16,17,18].

Elevated FFA concentrations may result from both an expanded fat mass with a reduced lipid storage capacity [3,4] and reduced peripheral clearance [2,19]. Impaired postprandial insulin-mediated suppression of whole-body adipose tissue lipolysis has been reported in insulin resistance, although lipolysis per unit fat mass is reduced [20,21]. Additionally, reduced suppression of the spillover of FA derived from lipoprotein lipase (LPL)-mediated TAG hydrolysis across adipose tissue during hyperinsulinemia has been shown in obese patients with type 2 diabetes mellitus (T2DM) compared with healthy controls [22], and in insulin resistant compared with insulin sensitive subjects [23]. An increased FFA supply to the liver may result in an increased very low-density lipoprotein (VLDL)-TAG output [22,23]. Our group has recently demonstrated that insulin resistance was associated with increased postprandial forearm muscle VLDL-TAG uptake in insulin resistant men [14].

Although substantial evidence indicates that disturbances in skeletal muscle lipid metabolism contribute to insulin resistance it is important to consider that IFG and/or IGT may represent distinct pathways towards T2DM, with impaired hepatic and peripheral insulin sensitivity as the predominant disorders in IFG and IGT subjects, respectively [24,25,26]. Here, we hypothesized that differential skeletal muscle FA handling may be one of the underlying mechanisms contributing to differences in peripheral insulin sensitivity between IGT and IFG subjects. To address this hypothesis, we used a dual stable-isotope approach in combination with arterio-venous measurements across forearm muscle, enabling differentiation between the metabolic fates of dietary *versus* endogenous FAs, as validated previously [27]. Furthermore, insulin sensitivity was assessed both by a hyperinsulinemic-euglycemic clamp and postprandial net skeletal muscle glucose uptake, and skeletal muscle biopsies were taken to investigate lipid metabolites, their fractional synthetic rate (FSR), and the transcriptional regulation of FA metabolism. The present study demonstrated that increased liver-derived TAG extraction and reduced lipid turnover of saturated FA, rather than DAG content, in skeletal muscle accompany the more pronounced insulin resistance in IGT *versus* IFG subjects.

## 2. Materials and Methods

The present study was conducted within the framework of a placebo-controlled double-blind randomized multi-center trial, carried out in two different centers in the Netherlands (PRESERVE study, VU University Medical Center, Amsterdam and Maastricht University Medical Centre^+^, Maastricht, the Netherlands) (*Clinical Trial Registration Number:* ISRCTN42786336) [28].

### 2.1. Subjects

Subjects were characterized by determining fasting and 2 h plasma glucose concentrations by an OGTT. 12 IFG (fasting plasma glucose (FPG) ≥6.1 mmol/L and <7.0 mmol/L (≥5.6 mmol/L and <7.0 mmol/L with a family history of T2DM), 2 h plasma glucose <7.8 mmol/L) and 14 IGT (2 h plasma glucose ≥7.8 mmol/L and <11.1 mmol/L, of which 7 combined IGT/IFG) Caucasian male and female subjects participated. Exclusion criteria were excess alcohol intake (>20 units/week), hepatitis and/or pancreatitis, abnormal liver and renal function tests (liver enzymes and serum creatinine >2 times the upper limits of normal, respectively), changes in body weight over the last 2 months (±3 kg), and medication known to affect glucose metabolism.

### 2.2. Study Design

All subjects underwent a hyperinsulinemic-euglycemic clamp. During a second visit, a postprandial study was performed to evaluate the contribution of dietary TAG (labeled with [U-^13^C]-palmitate) and endogenous TAG (VLDL-TAG and circulating FFA, both labeled with [^2^H_2_]-palmitate), to circulating lipids and skeletal muscle FA handling. Subjects were asked to refrain from drinking alcohol and to perform no strenuous exercise two days before a study day. In addition, they were asked to avoid intake of food products naturally enriched with ^13^C for 7 days before the study. The Medical-Ethical Committee of Maastricht University Medical Centre^+^ approved the protocol and all subjects gave written informed consent before participating in the study. The study was in line with the Declaration of Helsinki.

#### 2.2.1. Hyperinsulinemic-Euglycemic Clamp

A hyperinsulinemic-euglycemic clamp was performed to assess peripheral insulin sensitivity [29]. Briefly, a variable amount of 20% glucose and insulin (40 mU·m^-2^·min^−1^) was infused. The M-value was calculated based on the last 30 min (steady-state).

#### 2.2.2. Postprandial Study

Skeletal muscle metabolism and postprandial insulin sensitivity (net glucose uptake across skeletal muscle) was studied by measuring arterio-venous concentration differences across the forearm, adjusted for forearm blood flow (FBF). Cannulas were placed as previously described [30]. After collecting blood samples for background enrichment 90 min before meal ingestion (t-90), a continuous intravenous infusion of the stable isotope tracer [^2^H_2_]-palmitate (97% enrichment: Cambridge Isotope Laboratories, Andover, MA, USA) complexed to albumin was started (0.035 µmol·min^−1^·kg bw^−1^). Arterialized and deep venous blood sampling was started after 1 h of tracer infusion to allow for isotopic equilibration to occur. Blood samples were collected simultaneously at three baseline time points (t-30, t-15 and t0) and at several time points (t30, t60, t90, t120, t180 and t240) after consumption (within 5 min) of a standardized liquid high-fat mixed-meal (t0) (2.6 MJ, consisting of 61E% fat (35.5E% SFA, 18.8E% MUFA and 1.7E% PUFA), 33E% carbohydrate and 6E% protein) containing 200 mg [U-^13^C]-palmitate (98% enrichment; Cambridge Isotope Laboratories, Andover, MA, USA). Directly after blood sampling, FBF was measured by venous occlusion plethysmography [19].

Skeletal muscle biopsies (80–100 mg) were taken from *m. vastus lateralis* under local anesthesia using the Bergström technique with suction during fasting conditions and at the end of the postprandial period (t240) to determine skeletal muscle lipids and gene expression profiles (described in more detail below).

### 2.3. Biochemical Analyses

Plasma glucose concentration was determined using a hexokinase method (Gluco-quant, Roche Diagnostics, Mannheim, Germany). Glycated hemoglobin (HbA_1c_) was measured by cation exchange chromatography (Menarini Diagnostics, Florence, Italy; reference values: 4.3%–6.1%). Insulin concentration was quantified using an immunometric assay (Siemens Healthcare, Los Angeles, CA, USA). Plasma FFA (Wako NEFA C kit, Sopar Biochemicals, Koekelberg, Belgium) was analyzed using standard enzymatic techniques automated on a Cobas Fara centrifugal spectrophotometer (Roche Diagnostics, Basel, Switzerland). Plasma TAG (Sigma, St Louis, MO, USA), glycerol (EnzyPlus; Diffchamb, Västa Frölunda, Sweden) and lactate (ABX Diagnostics, Montpellier, France) were analyzed enzymatically on a Cobas Mira automated spectrophotometer (Roche Diagnostics, Basel, Switzerland).

To determine tracer enrichment in plasma FFA and TAG, we extracted total lipids from the plasma using chloroform/methanol 2:1. The FFA and TAG fractions were separated by thin-layer chromatography and derivatized to their methyl esters. Plasma fractions were analyzed for the ^13^C/^12^C ratio in a gas chromatography continuous-flow isotope ratio-mass spectrometer (Finnigan MAT-252 GC-IRMS, Bremen, Germany) and for enrichment of [^2^H_2_] (Finnigan Incos-XL GC-MS, Bremen, Germany). Plasma palmitate concentrations (µmol·min^−1^) were analyzed on an analytical GC with ion flame detection using heptadecanoic acid as internal standard.

### 2.4. Skeletal Muscle Lipids

Skeletal muscle biopsies were lyophilized and dissected free of extramyocellular lipid, blood, and connective tissue. Total lipids were extracted from 10 to 20 mg muscle sample using chloroform-methanol (2:1 vol/vol) and internal standards, and thereafter evaporated under nitrogen at 37 °C. The extracted lipids were separated into FFA, DAG, TAG, and PL by thin-layer chromatography [11]. Concentrations of FAs in the fractions were determined using an analytical GC. Stable isotope enrichment of the lipid fractions was determined by measuring the ^13^C-to-^12^C ratios on a GC-IRMS (Finnigan MAT-252).

### 2.5. Skeletal Muscle mRNA Expression

Expression of genes involved in TAG synthesis (DGAT 1 and 2: Diacylglycerol *O*-Acyltransferase 1 and 2, and GPAT: Glycerol-3-Phosphate Acyltransferase 1, mitochondrial) and *de novo* ceramide synthesis (SPTLC 1 and 2: serinepalmitoyl transferase 1 and 2, CERK: ceramide kinase, ASAH 1 and 2: *N*-acylsphingosine Amidohydrolase 1 and 2) were analyzed. Additionally, skeletal muscle gene expression of muscle carnitine palmitoyl transferase 1b (*m*CPT1: mitochondrial transport and oxidation FA), peroxisome proliferators activated receptor-α (PPARα: lipid transport, beta-oxidation), peroxisome proliferators activated receptor-δ (PPARδ: oxidative capacity), peroxisome proliferator-activated receptor-γ coactivator 1-α (PGC1α: mitochondrial biogenesis), NADH dehydrogenase 1 beta subcomplex 5 (NDUFB5: oxidative phosphorylation) and succinate dehydrogenase complex B (SDHB: oxidative phosphorylation) was determined by RT-PCR [11]. Gene expression was normalized relative to the geometric mean of the internal reference genes (β-actin and β-2-microglobulin). Accession numbers and RNA primer sequences can be found in Table 1.

### 2.6. Calculations

The net fluxes of metabolites across forearm muscle were calculated by multiplying the arterio-venous concentration difference by forearm plasma flow (FPF). FPF was calculated by multiplying FBF with (1-hematocrite/100). A positive flux indicates net uptake across forearm muscle, whereas a negative flux indicates net release from the tissue. Rate of appearance of FFA (Ra_FFA_) (µmol·kg^−1^·min^−1^) was calculated with Steele’s single poole equations for steady state and non-steady state for the fasting and postprandial state, respectively [31]. Labeled FFA and TAG concentrations were calculated as the product of tracer/tracee ratio (TTR) of [^2^H_2_]- and [U-^13^C]-palmitate and the palmitate concentration in FFA and TAG, respectively.

Fractional synthetic rates (FSR, %/h) of skeletal muscle TAG, DAG and PL during the postprandial phase were calculated by using skeletal muscle FFA as the precursor pool for lipid synthesis [11]. The degree of saturation of skeletal muscle TAG, DAG, PL and FFA (%) was calculated by dividing the sum of saturated FA by the total amount of FA in a fraction. ∆9-desaturase activity was estimated as the proportion palmitoleic acid (C16:1*n*-7) to palmitic acid (C16:0), and as the proportion oleic acid (C18:1*n*-9) to stearic acid (C18:0). Total skeletal muscle TAG, DAG, PL and FFA contents were estimated as the sum of the particular FA content of the assessed fraction. For comparison of postprandial responses, the area under the curve (AUC) for the total postprandial phase was calculated.

### 2.7. Statistical Analysis

Data are expressed as mean ± standard error of the mean (SEM). Differences in variables between groups were tested by ANCOVA with gender as covariate. Repeated-measures ANOVA was used to assess group differences in time (group by time effect) during the postprandial period. Previous studies have shown that the present sample size is sufficient to detect differences is skeletal muscle FA handling [14,32]. Statistical analyses were performed using SPSS version 16.0 (SPSS, Chicago, IL, USA). *P <* 0.05 was considered statistically significant.

## 3. Results

### 3.1. Subjects’ Characteristics

Subjects’ characteristics are summarized in Table 2. IGT and IFG subjects were matched for age and BMI. The M-value, reflecting peripheral insulin sensitivity, tended to be lower (*p* = 0.064) and 2 h glucose concentrations were significantly higher (*p* < 0.001) in IGT as compared to IFG subjects (Table 2).

### 3.2. Arterialized Metabolites, Forearm Muscle Metabolism and Forearm Blood Flow

**Figure 1:**
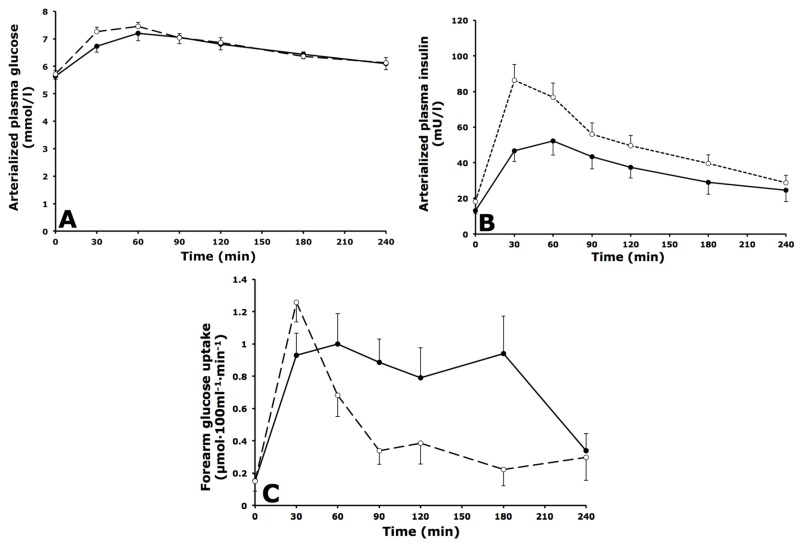
Fasting and postprandial glucose and insulin responses. (**A**) Arterialized plasma glucose concentrations; (**B**) Arterialized plasma insulin concentrations; (**C**) Net glucose uptake across forearm muscle during fasting (*t* = 0) and after consumption of a high-fat mixed-meal in IFG (black circles, solid lines) and IGT subjects (open circles, dashed lines). Values are mean ± SEM.

Plasma glucose concentrations were comparable between groups under fasting and postprandial conditions (Figure 1A). Fasting plasma insulin concentrations were not significantly different between groups but postprandial insulin concentrations were higher in IGT *versus* IFG subjects (AUC_0–4 h_: 51.3 ± 4.8 *vs.* 35.9 ± 5.1 mU·L^−1^·min^−1^ respectively, *p* = 0.042) (Figure 1B). Postprandial net glucose uptake across forearm muscle was significantly lower in IGT compared with IFG subjects (AUC_0–4 h_: 0.46 ± 0.05 *vs.* 0.79 ± 0.12 µmol·100mL^−1^·min^−1^ respectively, *p* = 0.018) (Figure 1C). Forearm blood flow and net glycerol fluxes were comparable between groups (Table 3).

### 3.3. Whole-Body and Forearm Muscle FFA Metabolism

#### 3.3.1. Whole-Body FFA Metabolism

**Figure 2:**
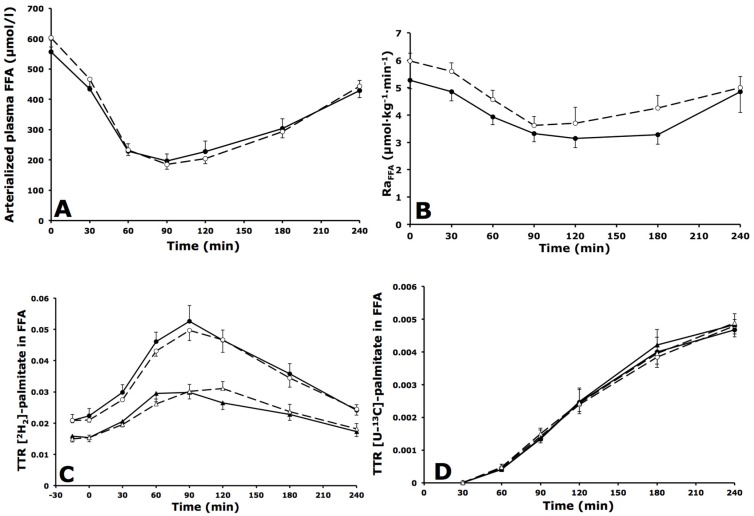
Fasting and postprandial whole body free fatty acid (FFA) metabolism. (**A**) Arterialized plasma FFA concentrations; (**B**) Rate of appearance (Ra) of FFA; (**C**) TTR [^2^H_2_]-palmitate concentrations in FFA during fasting (*t* = 0) and after consumption of a high-fat mixed-meal; (**D**) TTR [U-^13^C]-palmitate after consumption of a high-fat mixed-meal in IFG (black symbols, solid lines) and IGT subjects (open symbols, dashed lines). Circles represent arterialized plasma concentrations; triangles represent venous plasma concentrations. Values are mean ± SEM.

Fasting arterialized plasma FFA concentrations were comparable between groups. After meal consumption, arterialized plasma FFA concentrations decreased to the same extent in both groups, and returned to near-baseline values by the end of the postprandial period, with no significant differences between groups (Figure 2A). [^2^H_2_]-labeled palmitate was intravenously infused, mixed with the plasma FFA pool and reached steady-state concentrations during fasting measurements. The rate of appearance of FFA (Ra_FFA_) decreased after meal ingestion, indicating suppression of whole-body lipolysis with no differences between groups (Figure 2B). The TTR of [^2^H_2_]-palmitate in FFA was significantly higher in arterialized *versus* deep venous plasma at all time points in IGT and IFG subjects (both *p* < 0.001), indicating dilution of the [^2^H_2_]-tracer in the plasma FFA pool across forearm muscle (Figure 2C). The TTR of [U-^13^C]-palmitate in FFA (resulting from spillover of FA derived from chylomicron-TAG hydrolysis) was not significantly different in arterialized *versus* deep venous plasma in IGT and IFG subjects (*p* = 0.973 and *p* = 0.742, respectively) (Figure 2D).

#### 3.3.2. Forearm Muscle FFA Metabolism

Fasting and postprandial net plasma FFA extraction across forearm muscle was not different between groups. A consistent uptake of [^2^H_2_]-palmitate across forearm muscle was observed in both groups, with no significant differences between groups (Table 3).

### 3.4. Whole-Body and Forearm Muscle TAG Metabolism

#### 3.4.1. Whole-Body TAG Metabolism

**Figure 3:**
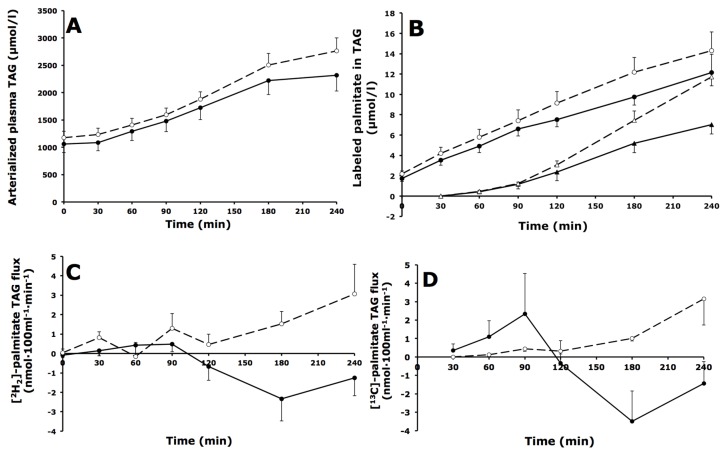
Fasting and postprandial whole-body triacylglycerol (TAG) metabolism. (**A**) Arterial plasma TAG concentrations; (**B**) [^2^H_2_]- and [U-^13^C]-palmitate concentrations in the plasma TAG fraction; (**C**) Net flux of [^2^H_2_]-palmitate TAG; (**D**) Net flux of [U-^13^C]-palmitate TAG across forearm muscle during fasting (*t* = 0) and after consumption of a high-fat mixed-meal in IFG (black symbols, solid lines) and IGT subjects (open symbols, dashed lines). Circles represent [^2^H_2_]-palmitate TAG; triangles represent [U-^13^C]-palmitate TAG. Values are mean ± SEM.

Arterialized plasma TAG concentrations were comparable during fasting and increased to the same extent after meal ingestion in IGT and IFG subjects (Figure 3A). The [^2^H_2_]-palmitate tracer could be measured in the plasma TAG fraction from the first baseline sample onward, reflecting incorporation of the intravenously infused tracer into VLDL-TAG (Figure 3B). Postprandial concentrations of [^2^H_2_]-palmitate in TAG were comparable in IGT and IFG subjects (AUC_0–4 h_: 8.9 ± 0.7 *vs.* 7.4 ± 1.1 mmol·L^−1^·min^−1^ respectively, *p* = 0.222) (Figure 3B). The [U-^13^C]-palmitate tracer, which was incorporated in the meal, appeared in the plasma TAG pool from 60 min after meal ingestion, representing chylomicron-TAG in the circulation (Figure 3B). During the postprandial period, [U-^13^C]-palmitate in TAG concentrations were not different between IGT compared with IFG subjects (AUC_0–4 h_: 4.3 ± 0.5 *vs.* 3.0 ± 0.5 mmol·L^−1^·min^−1^ respectively, *p* = 0.085).

#### 3.4.2. Forearm Muscle TAG Metabolism

Net TAG extraction across forearm muscle was not significantly different between IGT and IFG subjects (Table 3). The extraction of [^2^H_2_]-palmitate in TAG across forearm muscle was significantly higher (*p* = 0.043) in IGT compared with IFG subjects during the total postprandial period (Table 3, Figure 3C). Extraction of [U-^13^C]-palmitate in TAG across forearm muscle could be detected from 30 min onward, and was not different in IGT compared with IFG subjects (Table 3, Figure 3D). The fractional extraction of [U-^13^C]-palmitate derived from chylomicron-TAG hydrolysis (“entrapment”) was about 100% during the entire postprandial period in both groups, indicating a highly efficient forearm muscle uptake of FA derived from LPL-mediated TAG hydrolysis.

### 3.5. Intramuscular Lipid Metabolism

#### 3.5.1. Lipid Content

Skeletal muscle FFA content was comparable between both groups. However, TAG content was significantly higher (*p* = 0.025) in the IGT subjects whereas DAG (*p* = 0.031) and PL (*p* = 0.029) content were lower in IGT compared with IFG individuals (Table 4).

#### 3.5.2. Lipid Composition

The degree of saturation was significantly increased in the FFA fraction (*p* = 0.004) in IGT compared with IFG subjects, whilst TAG saturation was significantly lower (*p* = 0.017) and DAG saturation tended to be reduced (*p* = 0.059) in IGT subjects (Table 4). Also, the estimated ∆9-activity desaturase (ratio of C16:1*n*-7/C16:0) was significantly lower in the FFA fraction of IGT compared with IFG subjects (0.02 ± 0.01 *vs*. 0.07 ± 0.01, respectively, *p* = 0.016). In line, the FSR of skeletal muscle TAG tended to be lower in the IGT compared with the IFG group (0.13 ± 0.03 *vs*. 0.26% ± 0.07 %/h respectively, *p* = 0.073) (Table 4). These data indicate that a lower percentage of palmitic acid was directed towards the muscle TAG pool in IGT subjects. The FSR of skeletal muscle DAG, PL and FFA was not significantly different between groups (Table 4). Of note, the saturation of the different lipid pools within skeletal muscle was not significantly altered after meal intake as compared to baseline (data not shown).

### 3.6. Skeletal Muscle mRNA Expression

Fasting skeletal muscle mRNA expression of genes involved in FA transport, TAG and ceramide synthesis, and oxidative metabolism are shown in Table 5. Skeletal muscle gene expression of PGC1α (*p* = 0.027) and PPARα (*p* = 0.009) was significantly lower and gene expression of SDHB (*p* = 0.063) and CERK (*p* = 0.072) tended to be reduced in the IGT compared with the IFG subjects. Gene expression of the other genes was not significantly different between groups.

## 4. Discussion

The present study demonstrated an increased postprandial forearm muscle VLDL-TAG extraction and a reduced lipid turnover of, in particular, saturated FA in IGT compared with IFG subjects. These findings suggest that impairments in skeletal muscle FA handling may contribute to the more pronounced peripheral insulin resistance in individuals with IGT *versus* IFG.

Postprandial net glucose uptake across skeletal muscle was significantly lower in IGT compared with IFG individuals, despite higher plasma insulin concentrations. In line, insulin sensitivity, as determined by a hyperinsulinemic-euglycemic clamp, was reduced in IGT compared with IFG subjects. Taken together, these data clearly indicate that (postprandial) insulin sensitivity was lower in IGT than IFG subjects.

Disturbances in skeletal muscle FA handling have been implicated in the etiology of insulin resistance and T2DM [4,5,6,7,11,30]. Recently, our group has demonstrated a higher postprandial VLDL-TAG extraction by skeletal muscle in insulin resistant men compared with healthy controls, despite a similar TAG supply [14]. Furthermore, we have recently demonstrated that higher postprandial plasma TAG concentrations and increased net TAG extraction across forearm muscle was accompanied by decreased postprandial insulin sensitivity in subjects with impaired glucose metabolism compared with normal glucose tolerance [30]. The primary aim of the present study was to compare skeletal muscle fatty acid handling between IFG and IGT subjects by differentiating between dietary and endogenous FAs using dual stable isotope methodology, enabling detailed *in vivo* assessment of skeletal muscle FA metabolism [14,27]. Here, we demonstrated a significantly higher VLDL-TAG extraction in the more insulin resistant IGT as compared to IFG subjects, which is consistent with an increased muscle VLDL-TAG extraction in obese insulin resistant subjects [14]. Notably, measurements were not performed in the late postprandial phase, since it has been shown that dietary FA appear in the VLDL-TAG from 2 to 3 h after meal ingestion, making it difficult to separate chylomicron and VLDL-TAG in the late postprandial phase using the current dual isotope approach [33]. Therefore, we cannot exclude that the increased TAG extraction may also extend to chylomicron-TAG. One of the putative underlying mechanisms for the increased muscle TAG extraction may be an impaired inhibitory effect of insulin on skeletal muscle LPL action in the more insulin resistant IGT group.

Interestingly, in addition to increased TAG-derived FA uptake, we found pronounced disturbances in intramyocellar lipid partitioning in IGT subjects. The higher saturation of the intramuscular FFA pool and reduced saturation of the DAG and TAG pools in IGT subjects suggest increased retention of saturated FA in the muscle FFA pool. In accordance, despite higher muscle TAG concentrations, the FSR of the muscle TAG pool from dietary palmitate tended to be reduced in IGT subjects, which may point towards impaired lipolysis. Thus, these data hint towards a reduced partitioning of saturated FFA towards TAG synthesis in IGT subjects. Additionally, the expression of oxidative genes was reduced in the IGT group, suggesting that overall lipid turnover of saturated FFA is reduced in IGT *versus* IFG subjects. In accordance with our findings, a reduced fractional synthetic rate of intramuscular TAG together with higher TAG concentrations have recently been shown in obese prediabetic subjects as compared to controls [34]. Furthermore, a reduced incorporation of FFA into TAG in primary myotubes from obese individuals with type 2 diabetes has recently been shown [35]. Our data clearly extend these results by providing detailed insight into differences in skeletal muscle FA metabolism in relation to dietary and endogenous FA in IFG *versus* IGT subjects.

Several mechanisms may underlie impaired lipid turnover in IGT. The reduced TAG synthesis in IGT subjects may be related to a downregulation of lipogenic genes. However, genes involved in TAG synthesis (DGAT1-2 and GPAT) were not differentially expressed in skeletal muscle of both groups. Secondly, an increased content of saturated FFA in IGT subjects might be linked to an increased ceramide content and higher saturation in the long-chain fatty acyl-CoA, since increased levels of the saturated FA palmitate drive ceramide synthesis [36]. Notably, insulin sensitivity was inversely related to the saturation of ceramide and long-chain fatty acyl-CoA in obese humans [37]. Unfortunately, it was not possible to measure skeletal muscle ceramide levels in the present study. Nevertheless, expression of genes involved in *de novo* ceramide synthesis was not different in skeletal muscle of both groups in the present study. Finally, the increased saturation of the FFA pool may be related to a reduced activity of ∆9-desaturase. The present study showed that estimated skeletal muscle ∆9-desaturase activity was reduced in IGT compared with IFG subjects. Indeed, evidence is somewhat contradictory, but if anything, reduced rather than increased levels of ∆9-desaturase are positively associated with insulin resistance in humans [37]. Further research is required to unravel the mechanisms underlying impaired intramyocellular lipid turnover in IGT.

A limitation of the present study is that some of the IGT subjects also had IFG (combined IGT/IFG). Unfortunately, sample size did not allow separate analysis of isolated IGT subjects. Importantly, however, similar results were found when combined IGT/IFG subjects were removed from the IGT group. Secondly, we did not include a control group of healthy normal glucose tolerant subjects. We have previously demonstrated that subjects with impaired glucose metabolism show disturbances in skeletal muscle FA handling compared with normal glucose tolerant controls [30]. The aim of the present study, however, was to extend our previous results towards more detailed investigation of differences in dietary and endogenous skeletal muscle FA handling between subjects with IFG and IGT. Finally, we cannot entirely exclude that the differential muscle lipid handling in IFG *versus* IGT subjects may have been driven to some extent by differences in genetic background, since more subjects with a family history of T2DM were present in the IFG as compared to the IGT group (Table 2).

## 5. Conclusions

In conclusion, the present findings suggest that disturbances in skeletal muscle FA handling are more pronounced in IGT as compared to IFG subjects, and indicate that both increased skeletal muscle VLDL-TAG extraction and reduced lipid turnover of saturated FA, rather than accumulation of DAG, may contribute to insulin resistance.

## Figures and Tables

**Table 1:** Details of mRNA primer sequences.

Gene Symbol	Description	Primer Sequence	Accession Number
DGAT1	Diacylglycerol *O*-Acyltransferase 1	fw-TATTGCGGCCAATGTCTTTGC re-CACTGGAGTGATAGACTCAACCA	NM_012079.5
DGAT2	Diacylglycerol *O*-Acyltransferase 2	fw-GAATGGGAGTGGCAATGCTAT re-CCTCGAAGATCACCTGCTTGT	NM_001253891.1 NM_032564.4
GPAT GPAM	Mitochondrial glycerol-3-phosphate acyltransferase	fw-GATGTAAGCACACAAGTGAGGA re-TCCGACTCATTAGGCTTTCTTTC	NM_001244949.1/ NM_020918.5
SPTLC1	Serine palmitoyltransferase, long chain base subunit 1	fw-GGTGGAGATGGTACAGGCG re-TGGTTGCCACTCTTCAATCAG	NM_001281303.1 NM_178324.2 NM_006415.3
SPTLC2	Serine palmitoyltransferase, long chain base subunit 2	fw-TGCTCACGTATGTGGGGTATG re-GATTGGCCGATTCCAGTTGTC	NM_004863.3
ASAH1	Acid ceramidase I	fw-ATTGGCCCCAGCCTACTTTAT re-CCCTGCTTAGCATCGAGTTCAT	NM_001127505.1 NM_177924.3 NM_004315.4
ASAH2	Non-lysosomal ceramidase 2	fw-GTGCCTTTAACCCCAGAGTCT re-GTGCAGTCAGCTCGTCCAA	NM_001143974.1 NM_019893.2
CERK	Ceramide kinase	fw-TGTGCCTGTATCTGAGATCATCG re-TGCCGTCGTGCTCTCTTTAC	NM_022766.5
CPT1B	Carnitine palmitoyltransferase 1B (muscle)	fw-CGGGACAGGGGTAAGTTCTG re-TCTCGCAGGTCTGCTTTTGTG	NM_152246.2 NM_004377.3 NM_001145137.1 NM_001145135.1 NM_001145134.1 NM_152245.2
PPARGC1A	Peroxisome proliferator-activated receptor gamma coactivator 1-alpha	fw-TCTGAGTCTGTATGGAGTGACAT re-CCAAGTCGTTCACATCTAGTTCA	NM_013261.3
PPARA	Peroxisome proliferator-activated receptor alpha	fw-AAAAGCCTAAGGAAACCGTTCTG re- TATCGTCCGGGTGGTTGCT	NM_001001928.2 NM_005036.4
PPARD	Peroxisome proliferator-activated receptor delta	fw-AGAAGAACCGCAACAAGTGC re-CTCCCCTCGTTTGCAGTCAG	NM_001171818.1 NM_006238.4 NM_177435.2 NM_001171819.1
SDHB	Succinate dehydrogenase complex, subunit B	fw-CCACAGCTCCCCGTATCAAG re-TCGGAAGGTCAAAGTAGAGTCAA	NM_003000.2
NDFU5B	NADH dehydrogenase (ubiquinone) 1 beta subcomplex, 5	fw-GCTGCTCCTGTTCGACACA re-CTGCTAGTTCAGCTTGACCAAT	NM_001199958.1 NM_002492.3

**Table 2:** Subjects’ characteristics.

	IFG	IGT	*p*-Value
*n*	12	14	
Gender (male/female)	7/5	7/7	
Family history type 2 diabetes	10	6	
Age (years)	59.3 ± 1.7	58.6 ± 1.9	0.863
Weight (kg)	83.7 ± 4.1	90.3 ± 3.4	0.112
Height (cm)	170.6 ± 2.0	170.2 ± 2.4	0.816
BMI (kg/m^2^)	28.7 ± 1.1	31.2 ± 1.1	0.126
Waist/Hip ratio	0.98 ± 0.02	1.00 ± 0.02	0.329
Systolic BP (mmHg)	129 ± 3	135.1 ± 3	0.447
Diastolic BP (mmHg)	80 ± 2	83 ± 2	0.179
Fasting glucose (mmol/L)	5.9 ± 0.3	5.9 ± 0.2	0.786
2 h glucose (mmol/L)	5.3 ± 0.4	8.8 ± 0.8	<0.001
HbA1_C_	6.1 ± 0.1	6.0 ± 0.2	0.713
M-value (mg·kg^−1^·min^−1^)	4.1 ± 0.5	2.9 ± 0.3	0.064
Fasting insulin (mU/L)	12.2 ± 1.9	16.9 ± 2.1	0.093
Total body fat (%)	29.6 ± 2.0	33.6 ± 2.0	0.122
HDL cholesterol (mmol/L)	1.1 ± 0.1	1.2 ± 0.1	0.433
LDL cholesterol (mmol/L)	3.7 ± 0.2	3.6 ± 0.2	0.667
Triacylglycerol (mmol/L)	1.0 ± 0.1	1.4 ± 0.1	0.048

BMI, body mass index; BP, blood pressure. Values are mean ± SEM.

**Table 3:** Fasting and postprandial forearm blood flow and lipid metabolism.

	IFG (*n* = 12)	IGT (*n* = 14)	*p*-Value
**Forearm blood flow (mL·100mL^−1^·min^−1^)**			
Fasting	2.35 ± 0.19	2.59 ± 0.18	0.257
Postprandial (AUC_0–4 h_)	2.58 ± 0.22	2.85 ± 0.26	0.298
**Net fluxes across forearm muscle**			
Glycerol (nmol·100 mL^−1^·min^−1^)			
Fasting	−18.7 ± 7.8	−22.1 ± 19.0	0.978
Postprandial (AUC_0–4 h_)	−17.1 ± 7.3	−26.5 ± 15.4	0.753
FFA (nmol·100 mL^−1^·min^−1^)			
Fasting	50.3 ± 19.8	40.8 ± 37.6	0.851
Postprandial (AUC_0–4 h_)	37.1 ± 15.2	11.3 ± 19.8	0.379
[^2^H_2_]-palmitate FFA (nmol·100 mL^−1^·min^−1^)			
Fasting	1.59 ± 0.18	1.61 ± 0.19	0.917
Postprandial (AUC_0–4 h_)	2.45 ± 0.12	2.26 ± 0.14	0.335
TAG (nmol·100 mL^−1^·min^−1^)			
Fasting	30.3 ± 6.8	42.6 ± 18.9	0.656
Postprandial (AUC_0–4 h_)	104.7 ± 18.9	82.5 ± 15.9	0.204
[^2^H_2_]-palmitate TAG (nmol·100 mL^−1^·min^−1^)			
Fasting	−0.20 ± 0.17	0.01 ± 0.20	0.374
Postprandial (AUC_0–4 h_)	−0.57 ± 0.47	0.70 ± 0.24	0.043
[U-^13^C]-palmitate TAG (nmol·100 mL^−1^·min^−1^)			
Fasting	N/A	N/A	-
Postprandial (AUC_0.5–4 h_)	0.01 ± 0.32	0.77 ± 0.23	0.109

AUC, area under the curve (postprandial values expressed as AUC/min); N/A, not applicable; TAG, triacylglycerol. Values are mean ± SEM.

**Table 4:** Fasting skeletal muscle lipid content, composition and fractional synthetic rate after a high-fat mixed-meal.

	IFG (*n* = 12)	IGT (*n* = 14)	*p*-Value
**TAG**			
Total (mol/g dry weight)	178.1 ± 26.5	384.5 ± 91.8	0.025
Saturation (%)	35.6 ± 1.2	31.3 ± 1.1	0.017
MUFA (%)	47.0 ± 1.6	49.5 ± 1.7	0.190
PUFA (%)	17.4 ± 1.5	19.1 ± 1.9	0.562
FSR (%/h)	0.26 ± 0.07	0.13 ± 0.03	0.073
**DAG**			
Total (mol/g dry weight)	6.0 ± 1.0	3.0 ± 0.3	0.031
Saturation (%)	42.3 ± 1.9	36.4 ± 0.9	0.059
MUFA (%)	40.8 ± 1.4	48.7 ± 1.2	≤0.001
PUFA (%)	16.9 ± 1.2	14.9 ± 0.6	0.124
FSR (%/h)	0.39 ± 0.12	0.28 ± 0.06	0.439
**PL**			
Total (mol/g dry weight)	71.4 ± 3.4	59.2 ± 3.7	0.029
Saturation (%)	40.6 ± 1.3	39.7 ± 0.7	0.610
MUFA (%)	10.4 ± 0.4	11.4 ± 1.0	0.333
PUFA (%)	49.0 ± 1.3	48.9 ± 0.9	0.911
**FFA**			
Total (mol/g dry weight)	3.2 ± 1.0	2.2 ± 0.6	0.311
Saturation (%)	50.1 ± 3.5	68.0 ± 4.0	0.004
MUFA (%)	34.0 ± 2.9	19.7 ± 2.9	0.003
PUFA (%)	16.0 ± 2.6	12.3 ± 2.0	0.352

TAG, triacylglycerol; DAG, diacylglycerol; PL, phospholipids; MUFA, mono-unsaturated fatty acids; PUFA, poly-unsaturated fatty acids; FSR, fractional synthetic rate. Values are mean ± SEM.

**Table 5:** Fasting skeletal muscle gene expression.

Gene	IFG	IGT	*p*-Value
TAG synthesis			
DGAT1	1.79 ± 0.56	1.99 ± 1.03	0.203
DGAT2	0.25 ± 0.16	0.30 ± 0.25	0.195
GPAT1	1.79 ± 0.56	2.00 ± 1.03	0.477
Ceramide synthesis			
SPTLC1	1.36 ± 0.43	1.30 ± 0.47	0.181
SPTLC2	1.48 ± 0.38	1.42 ± 0.51	0.117
ASAH2	0.31 ± 0.18	0.50 ± 0.69	0.848
CERK	1.71 ± 0.88	1.31 ± 0.77	0.072
Oxidative metabolism			
*m*CPT1	2.01 ± 0.18	1.81 ± 0.18	0.434
PGC1α	0.63 ± 0.07	0.45 ± 0.04	0.027
PPARα	2.44 ± 0.22	1.70 ± 0.15	0.009
PPARδ	0.48 ± 0.03	0.43 ± 0.04	0.309
SDHB	2.00 ± 0.17	1.57 ± 0.13	0.063
NDFU5B	1.83 ± 0.14	1.66 ± 0.12	0.384

DGAT 1 and 2: Diacylglycerol *O*-Acyltransferase 1 and 2; GPAT: Glycerol-3-Phosphate Acyltransferase 1, mitochondrial;SPTLC1 and 2: serinepalmitoyl transferase 1 and 2; CERK: ceramide kinase, ASAH 1and 2: *N*-acylsphingosine Amidohydrolase 1 and 2; *m*CPT1, muscle carnitine palmitoyltransferase 1; PGC1α, proliferator-activated receptor-coactivator 1 alpha; PPARα, peroxisome proliferator-activated receptor alpha; PPARδ, peroxisome proliferator-activated receptor delta; SDHB, succinate dehydrogenase complex B; NDUFB5, NADH dehydrogenase 1 beta subcomplex 5. Values are mean ± SEM, expressed in arbitrary units.

## Abbreviations

The following abbreviations are used in this manuscript:

DAG	Diacylglycerol
FA	fatty acid
FBF	forearm blood flow
FFA	free fatty acids
FSR	Fractional Synthetic Rate
HbA1c	Glycated hemoglobin
IGM	Impaired Glucose Metabolism
IFG	Impaired Fasting Glucose
IGT	Impaired Glucose Tolerance
LPL	lipoprotein lipase
MUFA	mono-unsaturated fatty acid
OGTT	Oral Glucose Tolerance Test
PL	phospholipid
PUFA	poly-unsaturated fatty acid
Ra	rate of appearance
SFA	saturated fatty acid
TAG	Triacylglycerol
T2DM	type 2 diabetes mellitus
TTR	Tracer/Tracee Ratio
VLDL	very-low density lipoprotein
